# IL-1ra delivered from poly(lactic-*co*-glycolic acid) microspheres attenuates IL-1β-mediated degradation of nucleus pulposus *in vitro*

**DOI:** 10.1186/ar3932

**Published:** 2012-08-03

**Authors:** Deborah J Gorth, Robert L Mauck, Joseph A Chiaro, Bhavana Mohanraj, Nader M Hebela, George R Dodge, Dawn M Elliott, Lachlan J Smith

**Affiliations:** 1 Department of Orthopaedic Surgery, Perelman School of Medicine, University of Pennsylvania, 424 Stemmler Hall, 3450 Hamilton Walk, Philadelphia, PA 19104, USA; 2 Veterans Affairs Medical Center, 3900 Woodland Avenue, Philadelphia, PA 19104, USA; 3 Department of Biomedical Engineering, College of Engineering, University of Delaware, 125 E Delaware Avenue, Newark, DE 19716, USA

## Abstract

**Introduction:**

Inflammation plays a key role in the progression of intervertebral disc degeneration, a condition strongly implicated as a cause of lower back pain. The objective of this study was to investigate the therapeutic potential of poly(lactic-co-glycolic acid) (PLGA) microspheres loaded with interleukin-1 receptor antagonist (IL-1ra) for sustained attenuation of interleukin-1 beta (IL-1β) mediated degradative changes in the nucleus pulposus (NP), using an *in vitro *model.

**Methods:**

IL-1ra was encapsulated in PLGA microspheres and release kinetics were determined over 35 days. NP agarose constructs were cultured to functional maturity and treated with combinations of IL-1β and media conditioned with IL-1ra released from microspheres at intervals for up to 20 days. Construct mechanical properties, glycosaminoglycan content, nitrite production and mRNA expression of catabolic mediators were compared to properties for untreated constructs using unpaired Student's t-tests.

**Results:**

IL-1ra release kinetics were characterized by an initial burst release reducing to a linear release over the first 10 days. IL-1ra released from microspheres attenuated the degradative effects of IL-1β as defined by mechanical properties, glycosaminoglycans (GAG) content, nitric oxide production and mRNA expression of inflammatory mediators for 7 days, and continued to limit functional degradation for up to 20 days.

**Conclusions:**

In this study, we successfully demonstrated that IL-1ra microspheres can attenuate the degradative effects of IL-1β on the NP for extended periods. This therapeutic strategy may be appropriate for treating early-stage, cytokine-mediated disc degeneration. Ongoing studies are focusing on testing IL-1ra microspheres in an *in vivo *model of disc degeneration, as a prelude to clinical translation.

## Introduction

Intervertebral disc degeneration is strongly implicated as a cause of chronic low back pain, a condition that will affect up to 85% of people at some point during their lives [1,2]. Disc degeneration is a cascade that includes early changes to the cellular microenvironment and progresses over time to structural breakdown and functional impairment [3-5]. Anatomically, the intervertebral disc is composed of three distinct regions: the central gelatinous nucleus pulposus (NP), the surrounding fibrocartilaginous annulus fibrosus, and superiorly and inferiorly thin endplates of hyaline cartilage that interface with the vertebral bodies [5]. Changes to the NP are implicated in the initiation of the degenerative cascade. Decreasing proteoglycan content in the NP and the associated reduction in hydrostatic pressure impair the ability of the disc to evenly distribute and transfer compressive loads between the vertebrae [6,7].

Inflammation plays a key role in the progression of disc degeneration [8-11]. NP cells express the inflammatory cytokines interleukin-1 beta (IL-1β) and tumor necrosis factor-alpha (TNF-α), in particular, more highly in degenerate discs than in healthy discs [10]. The matrix composition of the NP, principally collagens and proteoglycans, is key to its mechanical function. Increasing levels of inflammatory cytokines in the degenerate disc have been associated with increased activity of catabolic proteases - including matrix metalloproteinases (MMPs) 1, 2, 3, 7, and 13 and a disintegrin and metalloproteinase with thrombospondin motifs 4 (ADAMTS-4) and others - which degrade these constituents [11-14]. Current therapies for disc degeneration, including conservative techniques such as physical therapy and surgical techniques such as spinal fusion [15], treat the symptoms of low back pain without addressing the underlying biological mechanisms and, as a result, fail to sustain or restore disc structure and function.

We recently described a three-dimensional (3D) agarose culture model of the NP that recapitulates key molecular, compositional, and biomechanical properties of the native tissue and is responsive to inflammatory stimulus [16]. In that study, NP constructs treated with IL-1β exhibited increased mRNA expression of inducible nitric oxide synthase (INOS), ADAMTS-4, and MMP-13 and decreased glycosaminoglycan (GAG) content and aggregate modulus [16]. These findings highlighted the importance of IL-1β as a mediator of intervertebral disc degeneration by linking upregulation of key catabolic molecules with compositional and biomechanical changes. Attenuating the catabolic effects of IL-1β in the NP may be an essential therapeutic step in slowing or reversing disc degeneration. Interleukin-1 receptor antagonist (IL-1ra) is an endogenous inhibitor of IL-1β *in vivo*, where it acts by competitively blocking the binding of IL-1β to IL-1 receptors on IL-1-responsive cells [17,18]. Whereas IL-1β is upregulated with disc degeneration, IL-1ra is not [9], resulting in an imbalance between catabolic and anabolic signaling.

Given the central role of IL-1 signaling in inflammatory diseases, considerable effort has been devoted to the development of IL-1ra for clinical applications [17,19-24]. IL-1ra has successfully been used clinically for the treatment of rheumatoid arthritis for a number of years [22-24]. IL-1ra has also been investigated as a potential treatment for osteoarthritis in animal and human clinical trials, and the results have been encouraging [19,20,25]. There are pathophysiological similarities between osteoarthritis and intervertebral disc degeneration, including chronic cytokine-mediated extracellular matrix degradation [26]. There are also phenotypic similarities between articular cartilage chondrocytes, which mediate the inflammatory cascade in osteoarthritis, and NP cells, which mediate the cascade in the disc [27]. As such, it has been suggested that IL-1ra may have potential for the treatment of disc degeneration [26]. This potential has been demonstrated by recent *in vitro *studies [16,28]. One study showed that IL-1ra delivered both directly in solution and by gene therapy to disc tissue explants was able to inhibit mRNA expression and activity of catabolic enzymes upregulated by IL-1β [28]. In addition, our recent study used an agarose 3D culture model of the NP to show that co-delivery of soluble IL-1ra could effectively inhibit IL-1β-mediated degradation at the molecular, compositional, and functional levels [16].

The avascular nature of the disc [29], in addition to the short terminal (serum) half-life and high therapeutic concentrations of IL-1ra [30], suggest that, *in vivo*, systemic delivery for treatment of disc degeneration is unlikely to be successful. Clinically, repeated intradiscal injections of IL-1ra necessary to sustain high local concentrations are impractical and may induce further degenerative changes via structural damage to the annulus fibrosus. Biodegradable poly(lactic-*co*-glycolic acid) (PLGA) microspheres have been used clinically for both local and systemic delivery of a number of small-molecule and protein-based therapies [31]. Given that these microspheres could be delivered directly to the disc space, they represent a novel mechanism for local, sustained, and controlled delivery of anti-inflammatory agents, including IL-1ra, to the disc. Therefore, the objective of this study was to advance this technology toward clinical application by investigating the therapeutic potential of IL-1ra-loaded PLGA microspheres for sustained attenuation of IL-1β-mediated NP degradation by using a 3D culture model.

## Materials and methods

**Figure 1:**
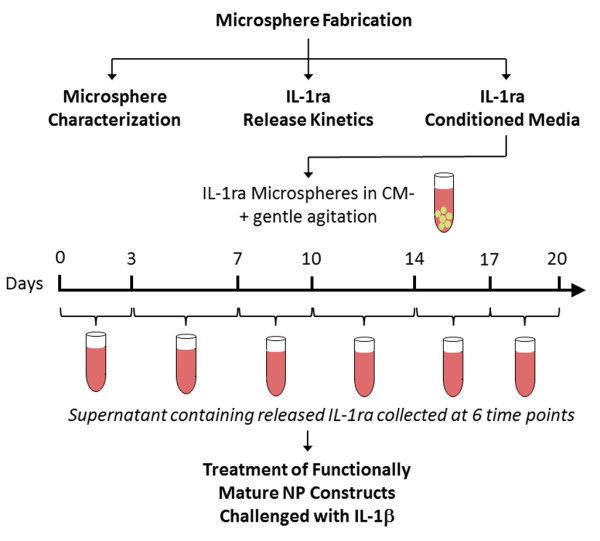
**Study design**. After fabrication, microsphere morphology was examined qualitatively by means of scanning electron microscopy. Interleukin-1 receptor antagonist (IL-1ra) release kinetics were quantified. IL-1ra conditioned media were generated by suspending microspheres in CM^- ^(chemically defined medium without transforming growth factor-beta 3) under gentle agitation. Media containing released IL-1ra were collected at the intervals indicated and used to treat functionally mature nucleus pulposus (NP) constructs that were challenged with IL-1β.

The sequence of experiments, from fabrication and characterization of IL-1ra microspheres to treatment of functionally mature NP constructs with IL-1ra microsphere-conditioned media and IL-1β, is illustrated in Figure 1.

### IL-1ra microsphere fabrication and characterization

We elected to use the clinically used form of IL-1ra, anakinra (Amgen, Thousand Oaks, CA, USA). With the exception of a single methionine residue on its amino terminus, anakinra is molecularly identical to native IL-1ra [21,32]. Anakinra (Kineret) was supplied as a syringe containing 100 mg in 0.67 mL of an aqueous solution buffered with 1.29 mg of sodium citrate, 5.48 mg of sodium chloride, 0.12 mg of disodium ethylenediaminetetraacetic acid, and 0.70 mg of polysorbate 80. Previous studies have used recombinant human IL-1ra (rhIL-1ra), rather than anakinra, to inhibit IL-1β-mediated degradation of NP [16,28]. Therefore, pilot studies were performed to demonstrate dosage-equivalent inhibition of IL-1β (10 ng/mL) by rhIL-1ra (R&D Systems, Minneapolis, MN, USA) and anakinra (both 100 ng/mL) for NP cells in two-dimensional culture (Figure S1 of Additional file 1). The use of anakinra (referred to simply as IL-1ra from this point forward) in place of rhIL-1ra in these studies enhances their translational potential given that IL-1ra already exists as a commercial product approved by the US Food and Drug Administration.

PLGA microspheres were fabricated by using the water-oil-water double-emulsion technique [33]. The initial water-oil emulsion was formed by combining 225 μL of IL-1ra (0.15 g/mL) with 1 mL of 75:25 PLGA (0.1 mg/mL) (Durect Corporation, Pelham, AL, USA) and sonicating the mixture at 20 kHz for 30 seconds (Ultrasonic Homogenizer 150 VT; BioLogics, Manassas, VA, USA). A total of 2 mL of 1% polyvinyl alcohol (PVA) (Sigma-Aldrich, St. Louis, MO, USA) was added, and the mixture was sonicated again at 20 kHz for 10 seconds to form the final water-oil-water emulsion. The double emulsion was then transferred to a 100 mL volume of 0.1% PVA and mixed with a magnetic stir bar for 3 hours. This microsphere-hardening phase was performed on ice to decrease the magnitude of the initial protein burst release [34]. The resulting microspheres were then washed three times with deionized water.

Samples for scanning electron microscopy (SEM) were collected by transferring 15 μL of microspheres in suspension directly onto SEM stages coated with carbon tape. The stages were stored at -20°C, allowing the phosphate-buffered saline (PBS) to sublimate. Samples were then sputter-coated with gold palladium and observed and imaged by using a scanning electron microscope (XL20; Philips, Amsterdam, The Netherlands) with a 10 kV accelerating voltage and a ×2,000 magnification. Remaining microspheres for all other experiments were then lyophilized and stored at -20°C.

### Quantification of IL-1ra release kinetics

To quantify IL-1ra release kinetics, microspheres were suspended in PBS at 500 μg/mL at 37°C under gentle agitation on a nutating platform. At 3- or 4-day intervals over the course of 20 days, microspheres were centrifuged at 4,000 revolutions per minute (rpm) for 10 minutes, the supernatant was collected, and microspheres were resuspended in fresh PBS. The IL-1ra content of supernatant collected for each interval was determined by using the bicinchoninic acid assay for total protein, and IL-1ra was used as the standard (Bio-Rad Laboratories, Hercules, CA, USA).

### Production of IL-1ra conditioned media

Media were conditioned with IL-1ra released from microspheres at intervals up to 20 days. IL-1ra microspheres were suspended in culture media (CM^-^) (defined below) at a concentration of 500 μg/mL at 37°C under gentle agitation. At 3, 7, 10, 14, 17, and 20 days, microsphere suspensions were centrifuged at 4,000 rpm for 10 minutes, and the conditioned media containing IL-1ra released during that interval were collected and frozen at -20°C. The microspheres were resuspended in culture media at 37°C for continued release of IL-1ra.

### Fabrication of functionally mature nucleus pulposus constructs

NP cell isolation and fabrication of functionally mature, 3D agarose constructs were carried out as described previously [16]. NP tissue was isolated via sharp dissection from the intervertebral discs of four bovine caudal spines purchased from a local slaughterhouse according to institutional guidelines. After dissection, the tissue was incubated overnight at 37°C in high-glucose Dulbecco's modified Eagle's medium (DMEM) with 2% penicillin/streptomycin/fungizone (PSF). Cells were isolated from the tissue by a two-step enzyme digestion consisting of pronase (1 hour in 2.5 mg/mL) followed by collagenase (4 hours in 0.5 mg/mL). After digestion, the cells were filtered through a 70 μm cell strainer before being expanded in monolayer in high-glucose DMEM containing 10% fetal bovine serum (FBS) and 1% PSF. Passage 2 cells were combined and suspended in a chemically defined medium that contained 10 ng/mL of transforming growth factor-beta 3 (TGF-β3) and that was designated CM^+^. The complete media formulation was published previously [16,35]. The cell suspension was mixed with an equal volume of 4% sterile, 49°C low-gelling temperature agarose to obtain a seeding density of 2.0 × 10^7 ^cells/mL. Gels were cast into a slab between two glass plates (2.25 mm in thickness), and individual constructs were punched out by using a 4 mm biopsy punch. NP constructs were cultured for 6 weeks in CM^+ ^before being transferred to chemically defined medium without TGF-β3 (designated CM^-^) for one additional week before treatments were commenced. Agarose (catalog # A4018) and collagenase were purchased from Sigma-Aldrich; DMEM, PSF (catalog # 15240), and FBS (lot # 769376) from Invitrogen (Carlsbad, CA, USA); pronase from Merck (Darmstadt, Germany); and TGF-β3 from R&D Systems.

### Treatment of nucleus pulposus constructs with IL-1β and IL-1ra conditioned media

IL-1ra conditioned media were thawed in a 37°C water bath immediately prior to the commencement of treatments. Constructs were allocated to each of eight treatment groups: CM^- ^alone (untreated), CM^- ^supplemented with recombinant human IL-1β (10 ng/mL), or IL-1ra conditioned medium collected at each of the six release intervals (0 to 3, 3 to 7, 7 to 10, 10 to 14, 14 to 17, and 17 to 20 days), also supplemented with IL-1β (10 ng/mL). This method of treatment was selected to evaluate long-term bioactivity of released IL-1ra since a direct co-culture of constructs and IL-1ra microspheres would require media changes every 3 days to maintain construct viability. This would result in removal of cumulatively released IL-1ra and require re-dosing with fresh IL-1β.

Constructs were harvested after 3 days of treatment, and biomechanical properties, GAG and collagen content, and mRNA levels of INOS, ADAMTS-4, MMP-13, IL-1β, IL-6, and Toll-like receptor 4 (TLR-4) were determined. Additionally, culture media were collected and analyzed for GAG and nitrite concentration. All results are presented as mean ± standard deviation. The properties for each of the treatment groups were compared with untreated and IL-1β-only treated constructs by using unpaired Student *t *tests. Significance was defined as a *P *value of less than 0.05.

### Mechanical properties

NP constructs (*n *= 5) from each treatment group were tested in confined compression (model 5542; Instron, Norwood, MA, USA). A detailed description of the testing apparatus was published previously [16]. Initially, a 0.02 N preload was applied for 500 seconds, followed by a stress relaxation test that consisted of 10% strain, calculated based on construct thickness following preload, applied at a rate of 0.05% per second, followed by a relaxation to equilibrium for 10 minutes. Aggregate modulus (H_A_) was calculated as the final equilibrium stress divided by the applied strain. Hydraulic permeability (k_o_) was calculated from the relaxation data by using linear biphasic theory, and material isotropy was assumed [36].

### Construct glycosaminoglycan and collagen content

GAG and collagen contents of NP constructs (*n *= 5) were calculated as described previously [16]. Briefly, after mechanical testing, samples were weighed and then were digested in papain at 60°C. The digests were assayed for sulfated GAG content by using the dimethylmethylene blue technique [37] and for collagen (after acid hydrolysis) by using the p-diaminobenzaldehyde/chloramine-T technique for hydroxyproline [38]. Collagen content was calculated, and a ratio of hydroxyproline to collagen of 1:10 was assumed [39]. GAG and collagen contents were normalized by construct wet weight.

### Glycosaminoglycan and nitric oxide released into the media

GAG content in the media was analyzed (*n *= 3) by using the dimethylmethylene blue assay and normalized per construct. Nitric oxide secreted into the media (*n *= 3) for each treatment group was determined by using the Griess reaction (Promega Corporation, Madison, WI, USA). The concentration of total nitrites was calculated from a sodium nitrite standard curve and expressed as micromole per construct.

### mRNA levels

Total RNA was isolated from NP constructs (*n *= 3) via two sequential extractions in TRIZOL/chloroform (Invitrogen) and was spectrophotometrically quantified (ND-1000; Nanodrop Technologies, Wilmington, DE, USA). Reverse transcription was performed on 1 μg of RNA with random hexamers by using a Superscript II kit (Invitrogen) in a 20 μL volume. We examined mRNA levels of six catabolic mediators: ADAMTS-4 (an aggrecanase), MMP-13 (a collagenase), and INOS (a cell stress marker); the cytokines IL-1β and IL-6; TLR-4; and the housekeeping gene glyceraldehyde 3-phosphate dehydrogenase (*GAPDH*). Primer sequences and sources [16,40] are provided in Table 1. Quantitative reverse transcription-polymerase chain reaction was performed by using SYBR Green reagents (StepOne Plus; Applied Biosystems, Carlsbad, CA, USA). Expression relative to GAPDH was calculated by using the comparative **cycle threshold **(CT) method [41]. Polymerase chain reaction efficiency was determined for each primer by running serial dilutions of control samples. For all primers and samples, amplification curves were parallel, and a single sharp dissociation peak was observed.

**Table 1:** Primer sequences used for real-time reverse transcription-polymerase chain reaction

Primer	Accession number	Direction (5' → 3')	Sequence	Melting temperature, °C	Product length, base pairs	Reference
*ADAMTS-4*	NM_181667.1	Forward	AACTCGAAGCAATGCACTGGT	54.96	149	[40]
		Reverse	TGCCCGAAGCCATTGTCTA	53.16		
*GAPDH*	NM_001034034.1	Forward	ATCAAGAAGGTGGTGAAGCAGG	54.61	101	[40]
		Reverse	TGAGTGTCGCTGTTGAAGTCG	55.17		
*INOS*	NM_001076799.1	Forward	GTAACAAAGGAGATAGAAACAACAGG	52.30	146	[16]
		Reverse	CAGCTCCGGGCGTCAAAG	55.52		
*IL-1β*	NM_174093.1	Forward	GAGCCTGTCATCTTCGAAACG	59.34	55	This paper
		Reverse	GCACGGGTGCGTCACA	60.32		
*IL-6*	NM_173923.2	Forward	GCTTCACAAGCGCCTTCACTCC	64.47	81	This paper
		Reverse	GACCCGGGGTAGGGAAAGCAGA	65.73		
*MMP-13*	NM_174389.2	Forward	TGGTCCAGGAGATGAAGACC	52.51	80	[40]
		Reverse	TGGCATCAAGGGATAAGGAA	50.22		
*TLR-4*	NM_174198.6	Forward	TGCGTACAGGTTGTTCCTAACATT	54.79	110	This paper
		Reverse	TAGTTAAAGCTCAGGTCCAGCATCT	55.50		

ADAMTS-4, a disintegrin and metalloproteinase with thrombospondin motifs 4; GAPDH, glyceraldehyde 3-phosphate dehydrogenase; IL-1β, interleukin-1 beta; IL-6, interleukin-6; INOS, inducible nitric oxide synthase; MMP-13, matrix metalloproteinase 13; TLR-4, Toll-like receptor 4.

### Effects of microsphere degradation products on nucleus pulposus properties

To examine the effects of PLGA microsphere degradation products on NP cells, blank microspheres (500 μg/mL, fabricated as above but without the addition of IL-1ra) were co-cultured with NP constructs for 3 days. Construct GAG, GAG released into the culture medium, and mRNA expression of INOS, ADAMTS-4, and MMP-13 were determined and compared with untreated and IL-1β (10 ng/mL)-only treated constructs.

## Results

### Microsphere morphology and IL-1ra release kinetics

**Figure 2:**
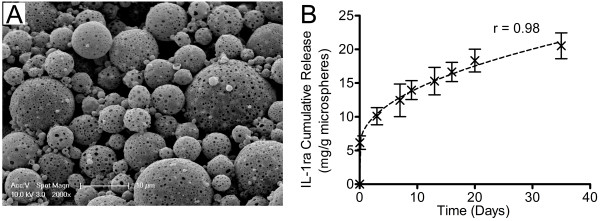
**Microsphere characterization and interleukin-1 receptor antagonist (IL-1ra) release kinetics**. **(a) **Microsphere morphology upon fabrication as viewed under scanning electron microscopy. **(b) **Cumulative release of IL-1ra from microspheres suspended in phosphate-buffered saline under gentle agitation over 35 days. Each data point is the mean ± standard deviation of three separate aliquots of microspheres in suspension. The dashed line represents a power series curve fit to the raw data. The power series curve is defined in the 'Microsphere morphology and IL-1ra release kinetics' section of the text.

Undegraded IL-1ra-loaded PLGA microsphere morphology, demonstrated by SEM, is shown in Figure 2a. Microsphere porosity was evident, and diameters were between 5 and 20 μm. The IL-1ra release profile (Figure 2b) was characterized by an initial burst release, decreasing to an approximately linear and sustained release over the initial 10 days. Release kinetics were found to be well approximated by a power series curve fit (r = 0.98) governed by the equation: $R=5.39\times T^{4.67\times 10^{-6}}+3.02\times T^{0.046}$, where *R *is cumulative release (expressed in milligrams of IL-1ra per gram of microspheres) and *T *is time (expressed in days). Cumulative release of IL-1ra in the media for each collection interval was estimated by using this equation (Table 2).

**Table 2:** IL-1ra concentration in IL-1ra microsphere conditioned media, estimated from a power series fit of release kinetics (Figure 2)

Media collection interval	Estimated concentration of IL-1ra, μg/mL
0 to 3 days	5.21
3 to 7 days	1.21
7 to 10 days	0.67
10 to 14 days	0.74
14 to 17 days	0.48
17 to 20 days	0.44

IL-1ra, interleukin-1 receptor antagonist.

### Attenuation of IL-1β-mediated degradation by IL-1ra released from microspheres

**Figure 3:**
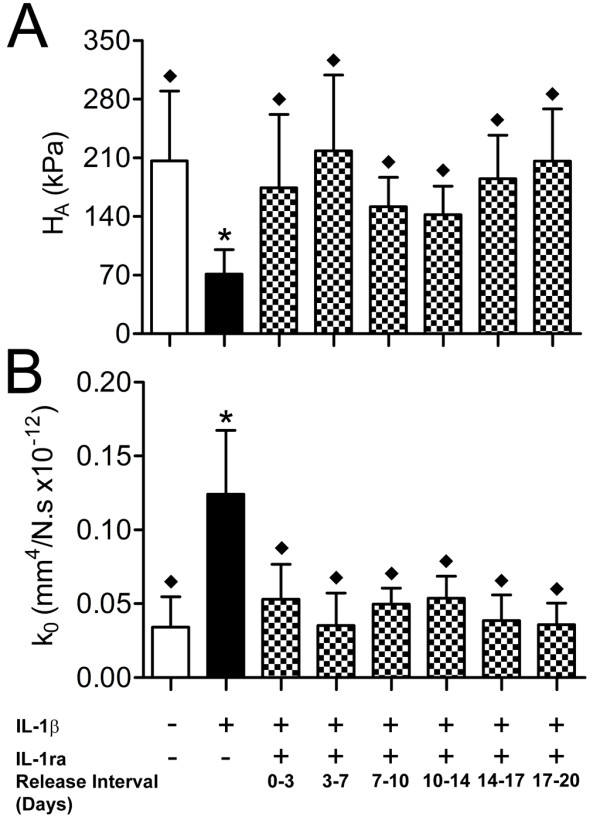
**Mechanical properties of nucleus pulposus constructs (*n *= 5) treated with IL-1β or IL-1ra conditioned media or both were evaluated in confined compression**. **(a) **Aggregate modulus (H_A_). **(b) **Hydraulic permeability (k_0_). Values are mean ± standard deviation. *P < 0.05 versus untreated constructs; ◆*P *< 0.05 versus IL-1β-only treated constructs. IL-1β, interleukin-1 beta; IL-1ra, interleukin-1 receptor antagonist; N.s., Newton second.

Mechanical properties of NP constructs treated with IL-1β or IL-1ra conditioned media or both are shown in Figure 3. IL-1β treatment alone resulted in functional degradation in terms of both construct modulus (35% of untreated constructs, *P *< 0.05, Figure 3a) and permeability (3.6-fold higher than untreated constructs, *P *< 0.05, Figure 3b). For constructs co-treated with IL-1β and IL-1ra conditioned media, modulus was significantly greater than for constructs treated with IL-1β alone and was not significantly different from untreated controls for all collection intervals. Similarly, permeability was significantly lower than for constructs treated with IL-1β alone and was not significantly different from untreated constructs for all collection intervals.

**Figure 4:**
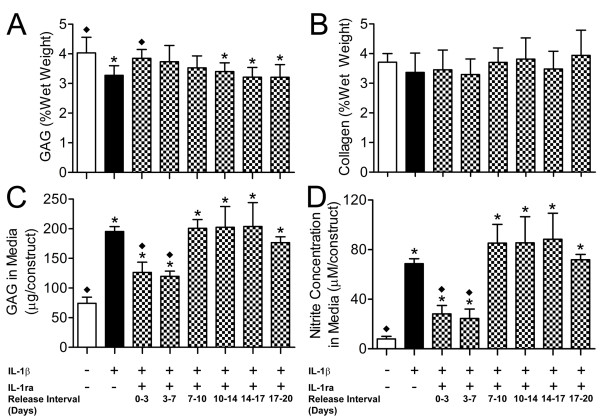
**Construct glycosaminoglycan (GAG) and collagen content and GAG and nitrites released into the treatment medium**. GAG and collagen content of nucleus pulposus (NP) constructs (*n *= 5), and GAG and nitrites released into the media (*n *= 3), after treatment with IL-1β or IL-1ra conditioned media or both, were determined. **(a) **GAG content of NP constructs. **(b) **Collagen content of NP constructs. **(c) **GAG released into the treatment medium. **(d) **Nitrites released into the treatment medium. Values are mean ± standard deviation. *P < 0.05 versus untreated constructs; ◆*P *< 0.05 versus IL-1β-only treated constructs. IL-1β, interleukin-1 beta; IL-1ra, interleukin-1 receptor antagonist.

When constructs were treated with IL-1β alone, GAG content was 81% that of untreated constructs (*P *< 0.05, Figure 4a). For constructs co-treated with IL-1ra conditioned media and IL-1β, GAG content was not significantly different from that of untreated controls for release intervals up to 10 days but was significantly lower at subsequent intervals. For co-treated constructs, GAG content was not significantly different from that of constructs treated with IL-1β alone for any interval except 0 to 3 days. There were no significant differences in construct collagen content for any of the treatment groups (Figure 4b).

GAG in the culture media for constructs treated with IL-1β alone was significantly higher (2.6-fold) than for untreated constructs (Figure 4c). For constructs co-treated with IL-1ra conditioned media and IL-1β, GAG in the media was significantly greater than that of untreated controls for all release intervals. However, media GAG for co-treated constructs was significantly lower than for constructs treated with IL-1β alone up to 7 days but was not significantly different at later intervals.

Nitric oxide in the culture media for each treatment group was measured as total nitrites by using the Griess reaction (Figure 4d). For constructs treated with IL-1β alone, media nitrite concentration was 8.7-fold higher than that of untreated controls (*P *< 0.05). For constructs co-treated with IL-1ra conditioned media and IL-1β, nitrite in the media was significantly greater than for untreated controls for all release intervals. However, similar to released GAG, media nitrite was significantly lower for co-treated constructs than for constructs treated with IL-1β alone up to 7 days but was not significantly different at later intervals.

**Figure 5:**
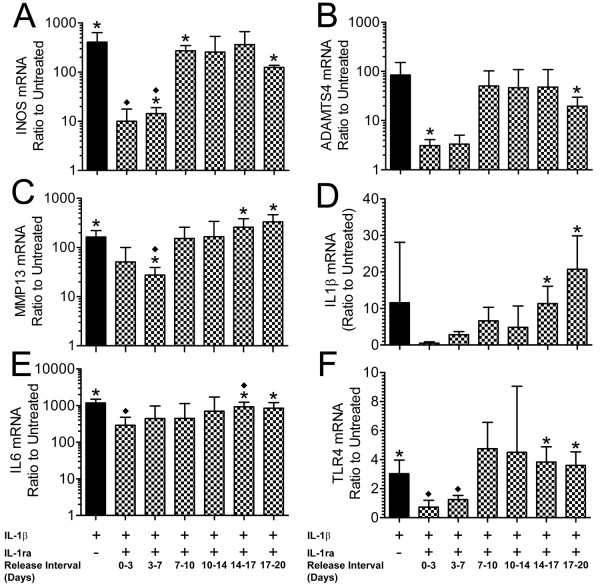
**mRNA expression levels of catabolic mediators**. mRNA expression levels for nucleus pulposus (NP) constructs (*n *= 3) after treatment with interleukin-1 beta (IL-1β) or interleukin-1 receptor antagonist (IL-1ra) conditioned media or both were quantified, normalized to glyceraldehyde 3-phosphate dehydrogenase (GAPDH), and expressed as a ratio to untreated constructs. **(a) **Inducible nitric oxide synthase (INOS). **(b) **A disintegrin and metalloproteinase with thrombospondin motifs 4 (ADAMTS-4). **(c) **Matrix metalloproteinase 13 (MMP-13). **(d) **IL-1β. **(e) **IL-6. **(f) **Toll-like receptor 4 (TLR-4). Values are mean ± standard deviation. *P < 0.05 versus untreated constructs; ◆*P *< 0.05 versus IL-1β-only treated constructs.

For each treatment group, mRNA levels were determined for six inflammatory mediators: INOS, ADAMTS-4, MMP-13, IL-1β, IL-6, and TLR-4 (Figure 5). For constructs treated with IL-1β alone, mRNA levels for INOS, ADAMTS-4, MMP-13, IL-1β, IL-6, and TLR-4 were 410-, 84-, 160-, 12-, 1,179-, and 3-fold higher, respectively, than for untreated constructs (all *P *< 0.05 except ADAMTS-4 and IL-1β). For constructs co-treated with IL-1ra conditioned media and IL-1β, mRNA levels for all six genes were higher than for untreated controls for all release intervals. However, the difference was an order of magnitude greater beyond 7 days, suggesting that attenuation of IL-1β signaling was less effective at these later time points (Figure 5). Similarly, mRNA levels for all six genes were lower than for constructs treated with IL-1β alone for the 0 to 3 and the 3 to 7 day IL-1ra release intervals but were not substantially different at later intervals. Significant differences are indicated in the figures. The failure to find significance for some comparisons is likely attributable to the combination of the variability of expression levels and the small sample size.

### Effects of microsphere degradation products

**Figure 6:**
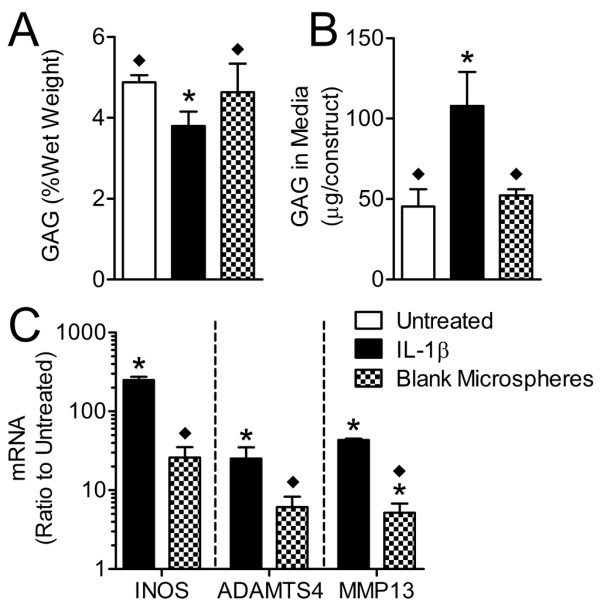
**Effect of microsphere degradation products on nucleus pulposus (NP) properties**. **(a) **Construct glycosaminoglycan (GAG) content. **(b) **GAG released into treatment media. **(c) **mRNA expression levels for inducible nitric oxide synthase (INOS), a disintegrin and metalloproteinase with thrombospondin motifs 4 (ADAMTS-4), and matrix metalloproteinase 13 (MMP-13) were normalized to glyceraldehyde 3-phosphate dehydrogenase (GAPDH) and expressed as a ratio to untreated constructs. Values are mean ± standard deviation. *P < 0.05 versus untreated constructs; ◆*P *< 0.05 versus IL-1β-only treated constructs.

There is some evidence that PLGA microsphere degradation products alone may elicit an inflammatory cellular response [42]. To examine the effects of these degradation products on NP cells, blank microspheres were co-cultured with NP constructs for 3 days and compared with the degradative effects of IL-1β. Construct GAG content and GAG released into the media for constructs co-cultured with blank microspheres were not significantly different from untreated constructs (Figure 6a, b). However, levels of mRNA for INOS, ADAMTS-4, and MMP-13 were elevated for constructs cultured with blank microspheres in comparison with untreated constructs (Figure 6c). These elevated mRNA levels, while at times large in terms of fold-change relative to controls, were significantly lower than for those changes that occurred for constructs treated with IL-1β (10%, 24%, and 12% of constructs treated with IL-1β for INOS, ADAMTS-4, and MMP-13, respectively; all *P *< 0.05).

## Discussion

In this study, we present evidence that IL-1ra delivered from PLGA microspheres can effectively attenuate IL-1β-mediated degradation of engineered NP constructs. IL-1ra is currently prescribed for the treatment of rheumatoid arthritis, and the treatment regime normally consists of daily self-administered subcutaneous injections [43]. This dosing frequency is necessary because of the short terminal half-life of the drug (approximately 4 to 6 hours) [30]. The predominantly avascular nature of the intervertebral disc [29] suggests that systemic delivery of IL-1ra is unlikely to be effective for treating inflammation in this tissue. Cells in the NP acquire nutrition via solute diffusion, primarily through the vertebral endplates [44]. It is possible that some drug may reach the NP via this route. However, given the short terminal half-life, the amount would most likely be small and lower than the therapeutic concentration. Thus, local delivery of IL-1ra into the NP would likely be necessary for effective treatment of NP inflammation. However, the tissues of the NP are subjected to continuous fluid flow because of mechanical loading and osmotic swelling, suggesting that the drug would rapidly diffuse away from the delivery site. Given that high-frequency injections to the disc are impractical, a sustained delivery vehicle would be required to make such a therapy clinically viable.

A key goal of sustained delivery techniques is achieving therapeutic levels of released factors over biologically relevant timescales. In one recent study, it was shown that IL-1ra released from PLGA microspheres could inhibit the stimulatory effects of IL-1β on tumor cell progression for at least 7 days [45]. Similarly, in the present study, we demonstrated that released IL-1ra comprehensively inhibited the degradative effects of IL-1β on NP constructs for up to 7 days in terms of composition, nitric oxide production, mRNA expression of catabolic factors and biomechanical properties. Beyond 7 days, the inhibitory effects of IL-1ra were limited to attenuation of construct GAG loss (up to 10 days) and of loss of biomechanical function (up to 20 days). Decreased overall efficacy at later time intervals may reflect both the decreasing concentration of released IL-1ra with time and potentially decreased bioactivity of the encapsulated protein. In a previous study, we demonstrated that 100 ng/mL of IL-1ra was sufficient to completely inhibit the degradative effects of 10 ng/mL of IL-1β on NP constructs [16]. As shown in Table 2, cumulative IL-1ra release was greater than 100 ng/mL for all intervals, suggesting that the decreased efficacy may be due to decreased bioactivity rather than insufficient delivery dose. We selected PLGA microspheres as the delivery vehicle for this study since they are biocompatible and easily optimized for different applications. The water-oil-water double-emulsion fabrication technique is an effective and well-characterized method for producing PLGA microspheres [31]. One limitation of this technique, however, is maintaining protein stability and activity during the fabrication process. The organic-aqueous interface can interfere with the hydrostatic mediated tertiary structure of proteins, and the mechanical cavitation forces present during emulsification can denature proteins [46]. After encapsulation, acid stress from the lactic and glycolic acid monomers from PLGA degradation can interfere with protein hydrogen bonding, leading to loss of protein activity [31]. For this study, we did not add additional stabilizing agents beyond those already present in the drug-buffering solution. In the future, additional protein stabilizers such as bovine serum albumin or additional acid buffers could be used to improve and maintain the bioactivity of IL-1ra after release. Another important consideration when evaluating the translational potential of this treatment is the fact that the concentration of IL-1β of 10 ng/mL used in this study likely far exceeds that which would likely be present *in situ *in the microenvironment of the degenerate disc, simulating an artificially harsh inflammatory environment.

To create microspheres for this study, we chose a PLGA with a high lactic acid ratio. The 75:25 PL/GA copolymer ratio used in this study is less hydrophilic than the more commonly used 50:50 ratio, leading to slower degradation [42,47]. This results in extended release and slower accumulation of acidic degradation by-products. Nevertheless, these degradation products, specifically lactic and glycolic acids, could be expected to lower the pH at the delivery site, placing stress on the local cell population. In their native environment, NP cells exist in relatively acidic conditions in part due to anaerobic glycolysis, a product of which is lactic acid [48], suggesting that they may be relatively resistant to acidic PLGA degradation products. There is also evidence that acidity in the NP is higher in symptomatic degenerate discs, which are the most likely candidates for therapeutic intervention [49]. Nevertheless, to evaluate these effects *in vitro*, we co-cultured our NP constructs with blank microspheres. While there was no significant effect on GAG content or release, there were increases in the mRNA levels of catabolic mediators, suggesting that the microsphere degradation products did have some effect. This highlights the importance of appropriate control groups to assess these effects when microspheres are tested *in vivo*.

Maintenance of biomechanical properties at later release intervals, despite increases in inflammatory mediators and decreased GAG content, was encouraging but unexpected and likely points to the importance of extracellular constituents other than GAG in contributing to mechanical function. Indeed, a previous study examining functionally mature chondrocyte-seeded agarose constructs cultured under free-swelling conditions found only a moderate correlation between GAG content and aggregate modulus (r^2 ^= 0.37) [50].

In this study, we found that treatment with IL-1β resulted in upregulation of TLR-4 mRNA expression. To our knowledge, this is the first time this finding has been reported for NP cells. While there are similarities between the cytoplasmic domains of IL-1 receptor and TLR-4, their extracellular domains are structurally distinct [51], suggesting that IL-1β does not signal directly through TLR-4. It is possible, however, that TLR-4 contributes to the inflammatory response indirectly by responding to endogenous ligands that are produced, for example, as a consequence of matrix catabolism.

A limitation of this study is our use of an engineered NP model, which lacks an exogenous biological response, mechanical loading, and other physiological factors. To address this limitation, ongoing work in our laboratory will seek to test this therapy in an *in vivo *model system. An additional limitation is our use of bovine cells. The adult bovine disc is considered to be an effective surrogate for the adult human disc for a number of reasons, including the absence of notochord-like NP cells and the large size leading to similar nutritional limitations for the cellular microenvironment. In this study, we specifically targeted IL-1β because this cytokine has been implicated as a primary mediator of matrix catabolism in disc degeneration. The inflammatory cascade is, however, a complex process mediated by a large variety of other cytokines, including IL-6 and TNF-α. Ongoing work will investigate therapies that specifically target these additional factors as well as the associated nuclear factor-kappa-B and mitogen-activated protein kinase signaling pathways [52].

## Conclusions

In this study, we provide evidence that IL-1ra delivered from PLGA microspheres can effectively attenuate IL-1β-mediated inflammatory changes in an engineered NP construct *in vitro*. Sustained delivery of anti-inflammatory agents may be very effective in preventing catabolic changes during early-stage disc degeneration but is unlikely to be sufficient to regenerate a painful and severely degenerate NP. A strength of this therapy may lie in a synergistic approach, in which sustained release of anti-inflammatory molecules is combined with complementary therapies for disc degeneration, such as those that augment and replace degenerate NP tissue, thereby halting the inflammatory cascade while simultaneously restoring disc height and mechanical function. Additional future work in our laboratory will focus on developing such synergistic approaches. More broadly, the techniques described in this study may have application for a wide variety of other musculoskeletal conditions characterized by acute or chronic inflammation.

## Abbreviations

3D: three-dimensional; ADAMTS-4: a disintegrin and metalloproteinase with thrombospondin motifs 4; CM: chemically defined medium; DMEM: Dulbecco's modified Eagle's medium; FBS: fetal bovine serum; GAG: glycosaminoglycan; GAPDH: glyceraldehyde 3-phosphate dehydrogenase; IL-1β: interleukin-1 beta; IL-1ra: interleukin-1 receptor antagonist; IL-6: interleukin-6; INOS: inducible nitric oxide synthase; MMP: matrix metalloproteinase; NP: nucleus pulposus; PBS: phosphate-buffered saline; PLGA: poly(lactic-*co*-glycolic acid); PSF: penicillin/streptomycin/fungizone; PVA: polyvinyl alcohol; rhIL-1ra: recombinant human interleukin-1 receptor antagonist; rpm: revolutions per minute; SEM: scanning electron microscopy; TGF-β3: transforming growth factor-beta 3; TLR-4: Toll-like receptor 4; TNF-α: tumor necrosis factor-alpha.

## Competing interests

The authors declare that they have no competing interests.

## Authors' contributions

LJS contributed to study conception and design, drafted the manuscript, and contributed to data analysis and interpretation. DJG performed cell culture, microsphere fabrication and characterization, mechanical testing, and biochemical assays and critically revised the manuscript for important intellectual content. JAC performed polymerase chain reaction experiments and critically revised the manuscript for important intellectual content. BM performed biochemical assays and critically revised the manuscript for important intellectual content. DME, RLM, GRD, and NMH contributed to study conception and design and critically revised the manuscript for important intellectual content. All authors read and approved the final manuscript.

## Supplementary Material

Additional file 1 **Figure S1**. **Bioactivity equivalence between recombinant human (rh) IL-1ra and anakinra**. NP cells were isolated as described in the methods, expanded to passage 2 and cultured in monolayer (high glucose DMEM, 10% FBS and 1% PSF) in 6-well plates (500,000 cells/well, *n *= 3 per condition). Twelve hours after plating, cells were treated for 24 hours with IL-1β (10 ng/ml) alone, IL-1β (10 ng/ml) + rhIL-1ra (100 ng/ml), or IL-1β + anakinra (100 ng/ml). Total RNA was extracted and INOS mRNA levels quantified via rt-PCR, normalized to GAPDH, and expressed as a ratio to untreated. For cells treated with IL-1β alone, INOS mRNA levels were significantly higher than untreated (p < 0.05, unpaired t-test). For cells co-treated with IL-1β and either rhIL1-ra or anakinra, INOS mRNA levels were not significantly different from untreated.; * p < 0.05 versus untreated; ◆ p < 0.05 versus IL-1β only treated. Click here for file
