# Supplementary figures and images for: IL-1ra delivered from poly(lactic-co-glycolic acid) microspheres attenuates IL-1β-mediated degradation of nucleus pulposus in vitro
Source: Arthritis Res Ther. 2012 Aug 3;14(4):R179. doi: 10.1186/ar3932 (PMC3580573; doi:10.1186/ar3932)

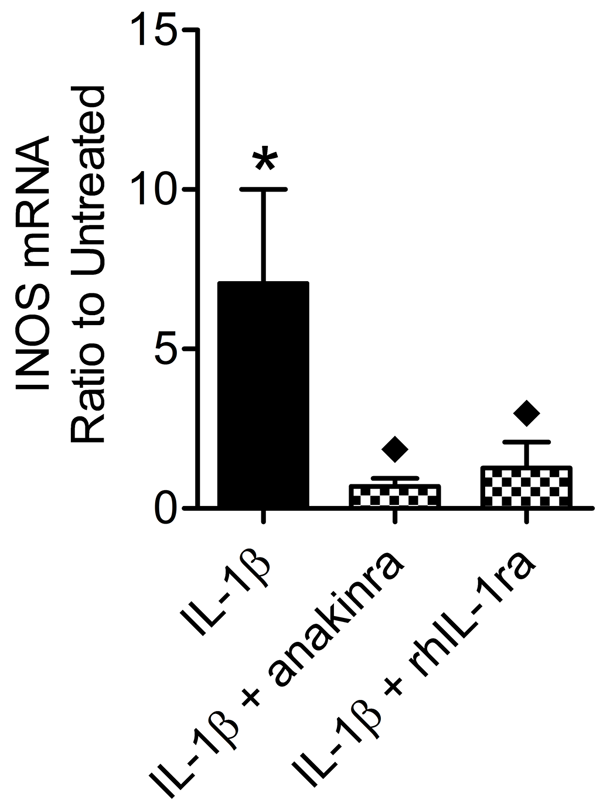

Supplement: Additional file 1 — Figure S1. Bioactivity equivalence between recombinant human (rh) IL-1ra and anakinra. NP cells were isolated as described in the methods, expanded to passage 2 and cultured in monolayer (high glucose DMEM, 10% FBS and 1% PSF) in 6-well plates (500,000 cells/well, n = 3 per condition). Twelve hours after plating, cells were treated for 24 hours with IL-1β (10 ng/ml) alone, IL-1β (10 ng/ml) + rhIL-1ra (100 ng/ml), or IL-1β + anakinra (100 ng/ml). Total RNA was extracted and INOS mRNA levels quantified via rt-PCR, normalized to GAPDH, and expressed as a ratio to untreated. For cells treated with IL-1β alone, INOS mRNA levels were significantly higher than untreated (p < 0.05, unpaired t-test). For cells co-treated with IL-1β and either rhIL1-ra or anakinra, INOS mRNA levels were not significantly different from untreated.; * p < 0.05 versus untreated; ◆ p < 0.05 versus IL-1β only treated. [file ar3932-S1.TIFF]
